# An advanced TMR sensor-based magnetrode for in vivo LFP magnetic field recording

**DOI:** 10.1038/s41378-026-01262-9

**Published:** 2026-05-13

**Authors:** Yi Wang, Jiahui Luo, Chenglong Zhang, Zhenhu Jin, Mixia Wang, Zhaojie Xu, Xinxia Cai, Jiamin Chen

**Affiliations:** 1 State Key Laboratory of Transducer Technology, Aerospace Information Research Institute, Chinese Academy of Sciences, Beijing, 100190 China; 2 School of Electronic, Electrical and Communication Engineering, University of Chinese Academy of Sciences, Beijing, 100049 China; 3 College of Materials Sciences and Opto-Electronic Technology, University of Chinese Academy of Sciences, Beijing, 100049 China

**Keywords:** Nanoscience and technology, Engineering

## Abstract

The detection and interpretation of brain signals are crucial for advancing brain-computer interface (BCI) technologies. Local field potential (LFP) signals, reflecting synchronized neuronal ensemble activity, offer insights into coordinated neural function. In this study, we present a miniaturized tunneling magnetoresistance (TMR)-based neural magnetrode optimized for in vivo LFP magnetic recording. The magnetrode achieves a magnetoresistance ratio (145%) and low-field sensitivity (16.59 %/mT), while maintaining low detection limits of 4.8 nT/√Hz at 1 Hz and 140 pT/√Hz at 1 kHz. Noise analysis revealed that reducing bias current and applying high-frequency AC excitation significantly suppress low-frequency 1/f noise. In vitro simulations validate LFP reconstruction capability, and in vivo experiments demonstrate a strong correlation (*r* = 0.857 ± 0.031, *p* < 0.01) between magnetic and electrical LFPs. Furthermore, in vitro durability tests in artificial cerebrospinal fluid demonstrated exceptional stability, with negligible signal drift (<0.4% variation in TMR ratio) over a 7-day period. This work establishes the TMR-based magnetrode emerges as a new potential tool for neural interface technologies, with implications real-time BCI and neuropathology research.

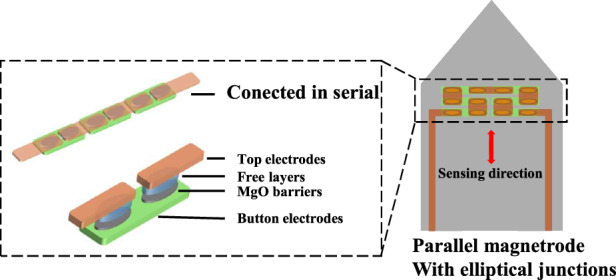

## Introduction

Brain-computer interfaces (BCIs) show great promise in diverse fields such as entertainment^1^, virtual reality^2^, disease diagnosis^3^, and medical rehabilitation^4^, thanks to ongoing advancements in science and technology. The core technology of BCIs lies in accurately interpreting brain signals, enabling direct communication between humans and external devices. These signals not only reflect complex neuronal activity but also encode vital information about consciousness, memory, and emotions^5^. As a result, detecting brain signals efficiently and accurately has become a central challenge in advancing BCI technology. The human brain contains approximately 86 billion neurons, interconnected by trillions of synapses, forming a highly complex neural network^6^. Over the past two centuries, neuroscience has made significant strides, particularly in understanding the structure and function of neural networks^7^. Neural activity generates signals at multiple scales: from the microscopic level of individual neuronal activity, to the mesoscopic level of local field potential (LFP) signals produced by the activity of neuronal ensembles, and to the macroscopic level of large-scale synchronized electrical activity in the brain. Detecting and analyzing these signals is crucial for unraveling how neural networks operate^8^.

As research increasingly focuses on large-scale neural ensembles, interest in LFP recordings has grown substantially, driven by their potential to advance clinical applications^9^. LFPs primarily reflect synchronized electrical activity of neuronal ensembles, which is mainly influenced by synaptic activity (e.g., excitatory and inhibitory postsynaptic potentials) near the implantation site^10^. These recordings are already being used to detect pathological changes associated with neurological diseases. For instance, in Parkinson’s disease (PD) patients, LFPs recordings have revealed abnormal synchronized activity in the 8–30 Hz frequency band within the striatum^11^. The study of LFPs also provides deeper insights into how neuronal ensembles coordinate during various cognitive tasks. Current LFP technology relies heavily on microelectrodes, particularly silicon-based electrode arrays like the “Utah electrode array” and “Michigan probes”^12–14^. These arrays improve control over recording site size and allow implantation across multiple brain regions. However, microelectrodes have several limitations for LFP signal recording. First, the electric field is highly dependent on the electrical conductivity of the tissue between neurons and electrodes, with electrical signals being significantly influenced by the variability in conductivity across different biological tissues. Second, measured potentials are always relative to a reference electrode, and the position and type of the reference electrode can significantly affect the recorded signals.

Neuronal activity generates not only electrical currents but also magnetic fields. Compared to electrical signals, magnetic signals propagate without distortion across different tissues due to the consistent magnetic permeability of various biological tissues. Furthermore, magnetic signal recording offer unique advantages, including non-contact operation, reference-free configuration, and vector signal detection. At the mesoscopic scale, which examines collective behavior in neuronal networks, magnetic sensors must be miniaturized and implantable to achieve close proximity to the source signals. Common magnetic sensors, such as superconducting quantum interference devices (SQUIDs^15,16^, atomic magnetometers^17–19^, and the nitrogen-vacancy diamond sensor^20^, face challenges in LFP detection due to bulkiness, structural complexity, and size limitations. In contrast, tunneling magnetoresistance (TMR) sensors, based on spintronics, emerge as a novel promising candidate for in vivo magnetic recording, owing to their high sensitivity, compact size, and low power consumption. Here, we present a new TMR-based neural magnetrode. We analyzed the noise power spectral density (PSD) of the magnetrode under different bias currents, revealing that reducing bias current and increasing excitation frequency effectively suppresses low-frequency noise. Furthermore, In vitro simulation experiments validated the magnetrode’s ability—paired with its interface circuit—to detect magnetic fields generated by LFPs. We then implanted the magnetrode into the hippocampus of rats, demonstrating its reliability by comparing recorded magnetic signals with simultaneously acquired electrical signals in vivo. Finally, to ensure the robustness of these findings and assess the device’s potential for chronic applications, we systematically evaluated its long-term stability and resistance to ionic corrosion in a simulated physiological environment. Collectively, these results establish the miniaturized TMR magnetrode as a robust and high-fidelity tool for future neural interface applications.

## Results

### Magnetrode structure and performance characterization

We designed and fabricated a miniaturized TMR-based magnetrode optimized for in vivo neural recording. The probe layout features an elliptical junction with an aspect ratio of 3 (short axis length is 15 μm), and a series-parallel connection of 12 MTJs, which can maintain relatively high sensitivity while achieving lower magnetic hysteresis^21^. To further clarify the rationale for selecting TMR technology for implantable neural magnetic sensing, we compared its key characteristics with other state-of-the-art magnetic sensing technologies (Table 1). Unlike bulky sensors that rely on large excitation coils or complex optical-thermal setups, the micron-scale dimensions and passive operation of the TMR device ensure high spatial resolution with minimal invasiveness, making it uniquely suitable for implantable bio-sensing.

**Table 1:** Comparison of TMR magnetrode with other magnetic sensors

Sensor type	Scale	Work temp.	System complexity	Implantability
SQUID^33^	System-scale	Cryogenic	**High:** Requires liquid helium cooling and magnetically shielded room	Low
OPM^18,34^	cm-scale	High	**High:** Vapor cells require heating and optical alignment	Low
NV Diamond^20,35^	mm-scale	Room Temp.	**Medium/High:** Requires complex optical excitation and microwave components.	Medium
TMR	μm-scale	Room Temp.	**Low:** Fully integrated device.	High

**Fig. 1:**
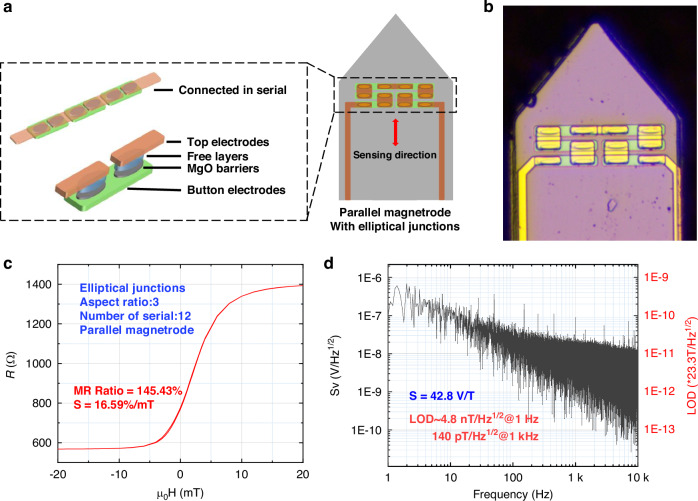
. **Magnetrode Structure and Performance Characterization**. **a** Schematic of the series-integrated elliptical-junction magnetrode; **b** Optical image of the fabricated device; **c** Magnetoresistance transfer curve (MR ratio: 145.43%, Sensitivity: 16.59%/mT); **d** Noise spectral density (LOD: 4.8 nT/Hz^1/2^ @ 1 Hz, 140 pT/Hz^1/2^ @ 1 kHz)

The schematic diagram of the magnetrode structure is shown in Fig. 1a, with the probe length designed to be 5 mm to match the depth of the rat hippocampus for subsequent in vivo experiments in rats. The width of the probe is 300 μm, which is mainly limited by the size of the MTJ. To achieve in vivo detection, the probe tip angle was designed to be 80°, ensuring penetration while minimizing damage to biological tissue.The optical image of the fabricated TMR magnetrode is presented in Fig. 1b.

The transfer curve of the magnetrode is shown in Fig. 1c, exhibiting a magnetoresistive ratio of 145% and a low-field sensitivity of 16.59%/mT. Figure 1d presents the noise performance of the magnetrode, achieving a low limit of detection(LOD) 4.8 nT/√Hz at 1 Hz and 140 pT/√Hz at 1 kHz. A comprehensive comparison in Table 2 confirms that the TMR-based magnetrode surpasses currently available implantable magnetrodes, offering a significant development in LOD for in vivo applications.

**Table 2:** Comparison of TMR magnetrode with other currently available implantable magnetrode

Work	MR Ratio (%)	LOD (nT/√Hz)			
		@1 Hz	@5 Hz	@10 Hz	@1 kHz
Valadeiro et al.^36^	4.0	-	54	~25	~3
Caruso et al.^37^	6.1	~22	~10	7	0.37
Klein et al.^38^	-	~30	~10	~8	1
This Work	145	4.8	2.7	1.8	0.14

“~” indicates values extracted from graphical data in the cited works, “-” indicates that the data was not reported in the cited works

### Noise performance of the magnetrode under different bias currents

Noise is inherent in all electronic devices^22^. For the magnetrode, its noise spectrum can be expressed as follows:

1 $${{\rm{S}}}_{{\rm{V}}}^{{\rm{Total}}}={\left({\rm{dB}}/{\rm{dV}}\right)}^{2}\left[{{\rm{S}}}_{{\rm{V}}}^{{\rm{shot}}}+{{\rm{S}}}_{{\rm{V}}}^{{\rm{elec}}-\frac{1}{{\rm{f}}}}+{{\rm{S}}}_{{\rm{V}}}^{{\rm{RTN}}}\right]+{{\rm{S}}}_{{\rm{V}}}^{{\rm{therm}}-{\rm{elec}}}+{{\rm{S}}}_{{\rm{V}}}^{{\rm{therm}}-{\rm{mag}}}+{{\rm{S}}}_{{\rm{V}}}^{{\rm{mag}}-\frac{1}{{\rm{f}}}}$$

Where ${{\rm{S}}}_{{\rm{V}}}^{{\rm{therm}}-{\rm{elec}}}{\rm{and}}{{\rm{S}}}_{{\rm{V}}}^{{\rm{therm}}-{\rm{mag}}}$ represent the thermal-electric noise and thermal-magnetic noise, respectively.${{\rm{S}}}_{{\rm{V}}}^{{\rm{shot}}}$ is the shot noise, which has a constant PSD across all frequency bands and is also referred to as white noise. ${{\rm{S}}}_{{\rm{V}}}^{{\rm{elec}}-\frac{1}{{\rm{f}}}}$ and ${{\rm{S}}}_{{\rm{V}}}^{{\rm{mag}}-\frac{1}{{\rm{f}}}}$ represent the electric 1/f noise and magnetic 1/f noise. ${{\rm{S}}}_{{\rm{V}}}^{{\rm{RTN}}}$ is the random telegraph noise^23^. All three are low-frequency noises. In the low-frequency region, 1/f noise is significantly higher than white noise, making it the key factor limiting the detection capability of the TMR-based sensor in low-frequency detection^24^. Since white noise is frequency-independent, the low-frequency noise of the TMR can be expressed as:

2 $${S}_{{{\rm{V}}}^{2}}=\frac{{\alpha }_{{\rm{H}}}{I}^{2}{R}^{2}}{{Af}}+k$$

Where k is a constant representing the sum of white noise, ${\alpha }_{{\rm{H}}}$ is the Hooge constant, I is the bias current, R is the device resistance, and A is the junction area.

**Fig. 2:**
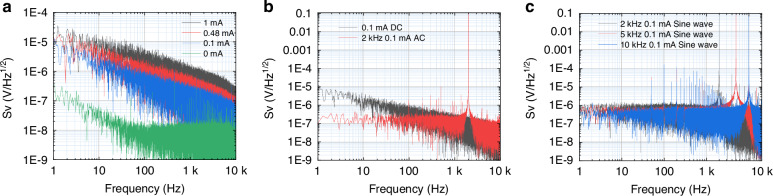
. **Effect of bias currents on Device Noise**: **a** PSD comparison at different DC bias; **b** PSD comparison between AC and DC bias; **c** PSD comparison at different bias frequencies

In the experiment, the bias current through the device was changed by adjusting the resistance value of the variable resistor. The experimental results are shown in Fig. 2a. As the bias current increases, the device noise also increases, which is consistent with Eq. 2, further verifying the physical mechanism that the increase in bias current leads to an increase in noise.

The results under AC bias are illustrated in Fig. 2b. Compared to DC bias, the AC-driven configuration shows significant suppression of low-frequency noise and a flatter noise spectrum. This is because the motion, size changes, and domain wall transitions of magnetic domains produce noise, which is particularly evident in the low-frequency range and has the characteristics of 1/f noise. When driving magnetic materials with a high-frequency AC bias, the magnetic domains can oscillate at higher frequencies, thereby changing the dynamic responses of the magnetic domains and achieving the effect of suppressing low-frequency noise^25^. However, significant power frequency noise is generated near the bias frequency and its harmonic components. Moreover, the magnitude of the AC bias frequency will directly affect the noise suppression effect. As shown in Fig. 2c, it can be seen that the higher the excitation frequency, the better the noise suppression, which plays an important role in improving the resolution of the TMR sensor. However, the bias frequency is limited by the TMR and data acquisition systems. Excessively high bias frequencies may adversely affect the sensitivity of the TMR and are limited by the data acquisition capability.

### In vitro simulation experiments

**Fig. 3:**
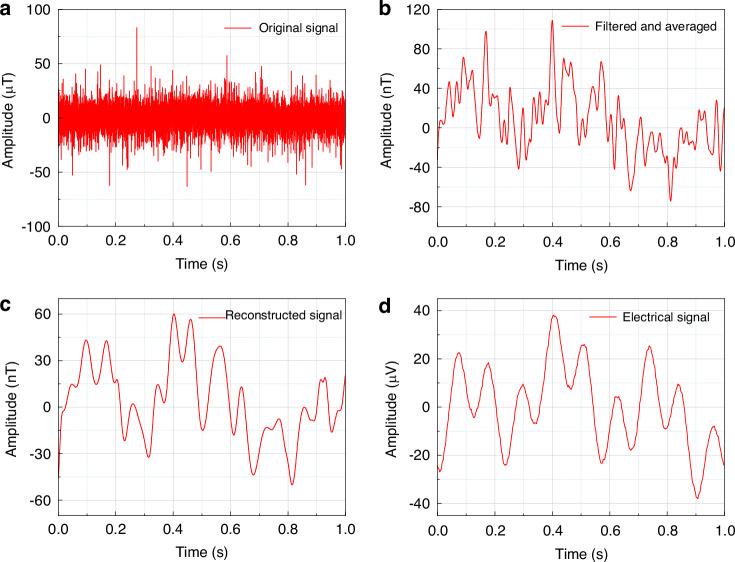
. **Detection results of the local field magnetic signal**: **a** Magnetic field raw signal; **b** Magnetic field signal after filter averaging; **c** Magnetic field signal after EMD reconstruction; **d** Local field potential signal

LFP signals are low-frequency potential fluctuations that reflect the synchronized activity of neuronal ensembles, originating from the postsynaptic currents of neurons within a local brain region. To verify the connectivity of the neural magnetrode with the interface circuit, a copper wire was employed to simulate the neuronal current source. While individual axons in the central nervous system typically exhibit diameters of 0.1–2 μm (unmyelinated) or 5–20 μm (myelinated), LFP signals differ from single-unit spikes as they represent the aggregate activity of neuronal ensembles. Consequently, the effective current source of an LFP signal has a spatial scale significantly larger than that of a single axon. Based on this, a 30 μm diameter copper wire was used as a reasonable physical model to represent localized neuronal ensembles. The wire was driven by a Digital Neural Signal Simulator (DNSS, Blackrock Microsystems), which is designed to supply simulated field potentials and action potentials. During the experiment, the copper wire and magnetrode were placed in a shielded bucket, and the data were collected after band-pass filtering and amplification in the range of 0.3–10 kHz. Subsequently, a 100 Hz low-pass filter was applied to reduce high-frequency noise interference, and a time window of 1 s was selected for multiple averaging of the signals to reduce random noise. Finally, the signals were reconstructed using the Empirical Mode Decomposition (EMD)^26^ to extract the magnetic signals corresponding to the LFP. Figure 3a–c show the original magnetic signal, the filtered and averaged signal, and the EMD-reconstructed signal within a 1-s time window, respectively. The original signal refers to the raw signal divided by the amplification factor and converted into a magnetic field according to the sensitivity of the magnetrode. Figure 3d shows the LFP signal with a period of 1 s generated by the electrical signal generator, which serves as a standard reference to verify the authenticity and validity of the magnetic signal. Comparison of Fig. 3c, d reveals that the EMD-reconstructed signal exhibits a high degree of consistency with the LFP signal, confirming the feasibility of the magnetrode and its interface circuit for detecting LFP magnetic signals.

### In vivo experiments

To further verify the feasibility of the neural magnetrode in in vivo detection, the neural magnetrode and microelectrode were simultaneously implanted into the hippocampal region of anesthetized rats. LFP represent the spatially weighted activity of neuronal populations and have an effective lateral recording radius of approximately 200–400 μm^27,28^. Based on this spatial extent, the separation between the magnetrode and the microelectrode recording sites was strictly controlled to be within 100 μm, ensuring that both probes sampled signals originating from the same neuronal population. Under this condition, the electrical signals detected by the microelectrode served as the standard reference signals to validate the reliability of the neural magnetrode in detecting magnetic signals in vivo. The microelectrode was connected to an electrophysiological detector. The interface circuit uses a Wheatstone half-bridge to effectively suppress the influence of environmental interference and temperature drift, ensuring the stability and reliability of the signals. After preamplification, the signals were filtered and secondarily amplified by the SR560, then converted from analog to digital by a data acquisition card, and transmitted to a PC for subsequent processing. To reduce the interference of the external environment on the LFP magnetic signals, the anesthetized rats with implanted devices were transferred to a shielded bucket for experimentation. To ensure the comprehensiveness and representativeness of the analysis, four segments of 100 s magnetic signals were recorded, which were then compared with the electrical LFP signals to verify the authenticity and validity of the magnetic signals. First, a fourth-order Butterworth band-pass filter was applied to remove frequency components unrelated to the signal. Then, the Welch method was used to calculate the PSD of each segment of the filtered magnetic signal, which was normalized for comparison with the electrical signal. Normalization helps to eliminate amplitude differences between different data segments, making the comparison more accurate.

**Fig. 4:**
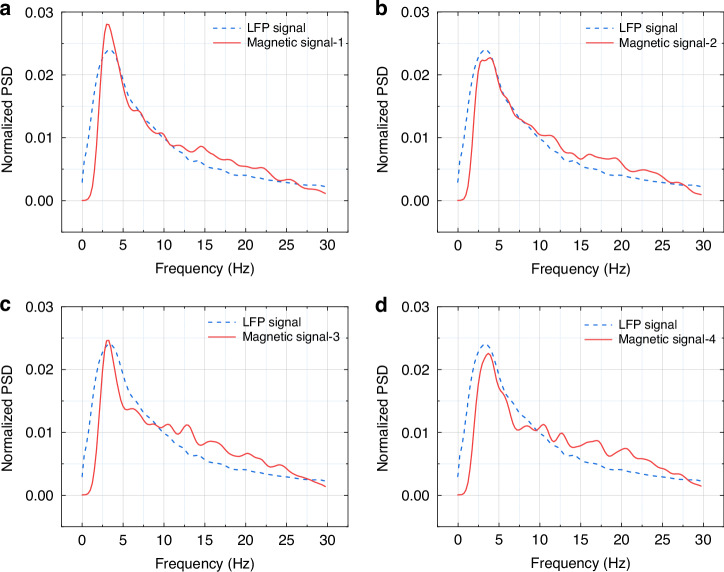
. **Experimental test results on live animals**: **a**–**d** Normalized PSD comparison between magnetic and LFP signals

The normalized PSD results of the electrical and magnetic signals are shown in Fig. 4, in which represent the normalized magnetic signal results for four segments of 100 s magnetic signals, respectively. The relative signal strengths of the two signals are similar at different frequencies, especially near the peak frequencies, where both signals reach significant peaks at similar positions. This intuitively demonstrates the correlation between the two signals, indicating that both signals are responses to the same source, namely neuronal activity.

**Fig. 5:**
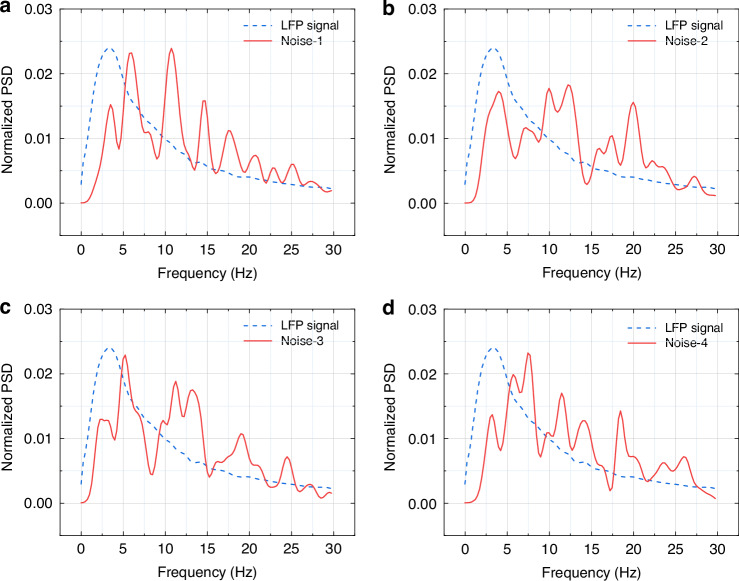
. **Experimental test results on control group**: **a**–**d** Normalized PSD comparison between noise and LFP signals

To further verify the reliability of the magnetic signals and ensure they are generated by LFP rather than noise, the noise results from the same magnetrode in the shielded bucket before implantation were used as the control group. Four segments of noise data were randomly selected, and their normalized PSD was calculated using the same method as for the magnetic signals, then compared with the LFP signals. The results are shown in Fig. 5, in which a–d represent the noise results of four different segments, respectively. It can be seen that the noise had a low degree of match with the LFP and no obvious correlation.

To quantify the correlation between magnetic signals, noise, and LFP signals, the Pearson Correlation Coefficient (PCC) was introduced as a measure. PCC is a widely employed statistical indicator for measuring the linear correlation between two variables, with a value range of [−1, 1], where 1 indicates a perfect positive correlation, −1 indicates a perfect negative correlation, and 0 indicates no correlation^29^. The calculation formula is:

3 $${\rm{r}}=\frac{{\sum }_{i=1}^{n}\left({X}_{i}-\bar{X}\right)\left({Y}_{i}-\bar{Y}\right)}{\sqrt{{\sum }_{i=1}^{n}{\left({X}_{i}-\bar{X}\right)}^{2}}\sqrt{{\sum }_{i=1}^{n}{\left({Y}_{i}-\bar{Y}\right)}^{2}}}\,$$

**Fig. 6:**
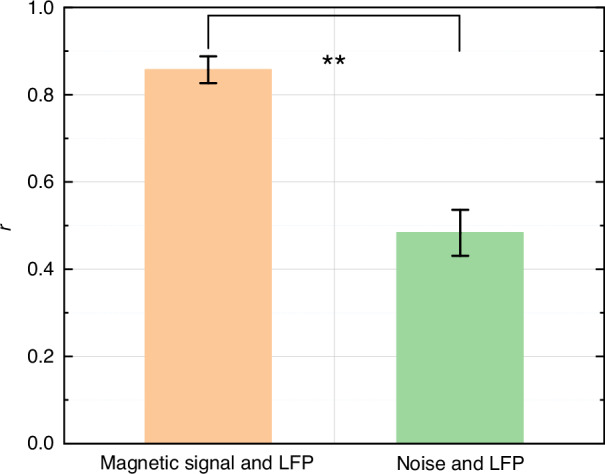
**Correlation analysis between magnetic signals, noise and LFP signals**. **P < 0.01

Where r is the correlation coefficient, *n* is the sample size, and *X* and *Y* are the values of the two variables. The normalized PSDs of the magnetic signals, noise, and LFP signals were calculated, and the results are shown in Fig. 6. The PCCs r between the magnetic signal PSD and the LFP PSD for the four independent 100-s segments were 0.8883, 0.8832, 0.8269, and 0.8307, yielding a mean value of 0.8573 ± 0.0307, indicating a stable and reproducible spectral correlation across time.In contrast, when the magnetic noise signal was used as a control, the corresponding correlation coefficients were 0.4578, 0.4660, 0.5575, and 0.4520 (mean ± SD = 0.4833 ± 0.0528), which are significantly lower, confirming that the observed correlation is not caused by background noise.

To rigorously validate this difference, a paired *t*-test was performed on the correlation coefficients of the normalized PSDs of the magnetic signals, noise signals, and LFP signals. The paired *t*-test is a statistical method used to compare the differences between two sets of related or matched samples. The results of the paired *t*-test showed a *P* value of less than 0.01 (*P* < 0.01), indicating that the correlation coefficient between the magnetic signals and LFP signals is significantly higher than that between the noise and LFP signals. This statistical result further confirmed that the detected signals were indeed magnetic signals generated by neuronal activity, rather than noise.

To further evaluate the frequency specificity of the detected magnetic signals, PCCs were calculated between the PSDs of the magnetic signal and the LFP within standard physiological frequency bands. High spectral correlation was observed in the Delta (1–4 Hz, *r* = 0.96 ± 0.01), Theta (4–8 Hz, *r* = 0.97 ± 0.03), and Beta (13–30 Hz, *r* = 0.93 ± 0.03) bands, while the Alpha band (8–13 Hz) exhibited moderate-to-high correlation (*r* = 0.79 ± 0.19). The exceptionally high correlation in the Theta band is particularly relevant, as theta oscillations constitute the dominant component of hippocampal LFP activity. This result indicates that the TMR magnetrode is capable of capturing the principal low-frequency neural dynamics that govern hippocampal population activity.

### Biostability of magnetrode under physiological conditions

Following the successful demonstration of acute in vivo neural recording, it is essential to evaluate the biostability of the TMR-based magnetrode, as the intracranial environment is chemically aggressive due to its high humidity and ionic concentration. Degradation of the encapsulation layer could potentially result in electrolyte penetration, sensor corrosion, or baseline drift, thereby compromising the fidelity and reliability of neural recordings.

To assess the durability of the magnetrode under simulated physiological conditions, an in vitro immersion test was performed. The fabricated devices were immersed in a standard artificial cerebrospinal fluid (aCSF) solution and maintained at a constant physiological temperature of 37 °C using a thermostatic incubator. The R-H curves were characterized at fixed time points (Day 0, Day 3, and Day 7). During each measurement session, key performance metrics—including the TMR ratio and sensitivity—were extracted to quantitatively evaluate device stability.

**Fig. 7:**
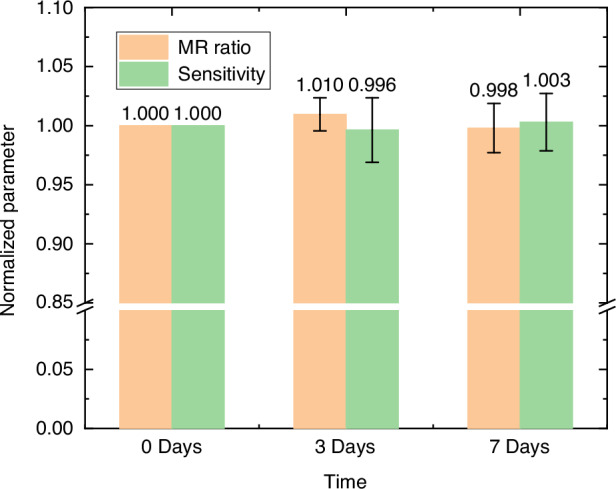
Stability of TMR magnetrode during in vitro aCSF immersion at 37 °C

Figure 7 summarizes the normalized variations of these parameters over the 1-week immersion period. The results demonstrate excellent robustness of the encapsulated magnetrodes: the TMR ratio exhibited a fluctuation of less than 0.4% relative to its initial value, while the sensitivity varied by less than 2%. This negligible performance degradation confirms that the encapsulation layer provides effective isolation against ionic corrosion, ensuring that the device maintains high structural integrity and signal reliability during the operational window of acute experiments.

## Discussion

In this work, we fabricated a high-sensitivity, low-noise, and compact TMR-based magnetrode for LFP magnetic recording. The neural magnetrode achieved low detection limits of 4.8 nT/√Hz at 1 Hz and 140 pT/√Hz at 1 kHz. By varying the bias current and its frequency, we investigated the impact of bias current on noise, confirming the physical mechanism behind the noise. Subsequently, we conducted LFP signal simulation experiments to validate the performance of the magnetrode. The recorded signals from the magnetrode showed a high degree of consistency with the LFP signals, preliminarily demonstrating the feasibility of the magnetrode and its interface circuit for detecting LFP magnetic signals. Finally, the fabricated neural magnetrode was implanted into the hippocampal region of rat brains for LFP magnetic signal recording. By comparing the normalized magnetic signals with LFP signals and calculating their correlation coefficients, we confirmed the feasibility and great potential of our magnetrode in LFP magnetic signal recording. Furthermore, our stability tests demonstrated negligible drift in TMR ratio and sensitivity over 7 days, indicating robust encapsulation against ionic corrosion. This work establishes the TMR-based magnetrode as a novel, promising tool for high-resolution neural recording, with potential applications in BCIs and pathophysiology studies of neurological disorders.

## Materials and methods

### Magnetrode fabrication

The magnetic tunnel junction (MTJ) was fabricated on a Si/SiO_2_ substrate. The multilayer stack incorporates a dual-pinning structure with the following configuration: Ta/Ru/Ta/Ru/Ta/Ru/PtMn/CoFe/Ru/CoFeB/MgO/CoFeB/NiFe/IrMn/Ru/Ta/Ru. The synthetic antiferromagnetic (SAF) multilayer structure, composed of CoFe/Ru/CoFeB trilayers, was designed to stabilize the magnetization direction of the reference layer while minimizing stray field interference on the free layer^30^. The composite free layer, consisted of CoFeB/NiFe/Ru/IrMn, effectively reduces the saturation field while achieving a higher TMR ratio, thereby enhancing sensitivity^31^. Benefiting from the different Néel temperatures of the two antiferromagnetic layers, the orthogonalization of the easy axes between the free layer and the pinned layer can be achieved through two annealing steps before and after microfabrication, resulting in a linear response^32^. A Cr/Au (30/200 nm) contact bilayer was deposited on the top of the MTJ by magnetron sputtering. Subsequently, a SiO_2_/Si_3_N_4_ (200/200 nm) passivation layer was deposited on the magnetrode surface by ICP-CVD to protect and enhance its biocompatibility.

### Experimental setup

**Fig. 8:**
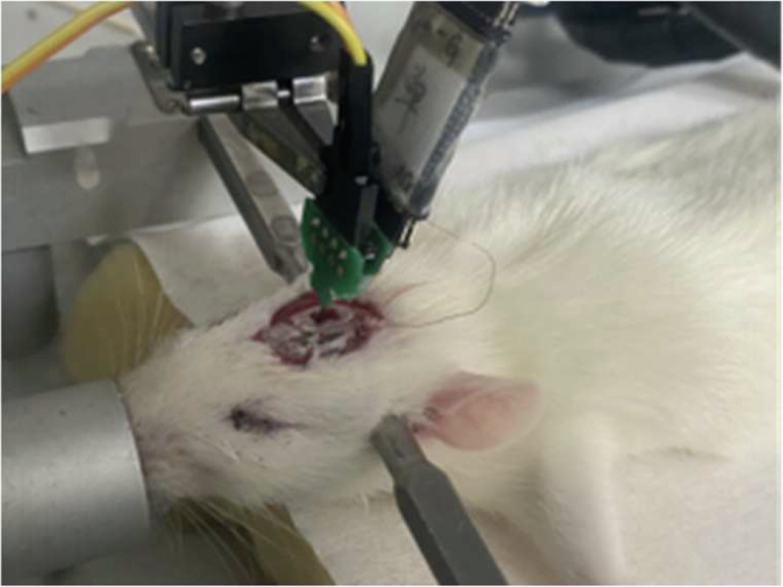
Surgical implantation of dual devices into the rat hippocampus

Due to the small size of the rat brain region and the constraints of the double locator implantation position, the neural magnetrode and microelectrode were bonded together, with their tips positioned as closely as possible. In this experiment, it was assumed that both devices detected signals from the same source. Rats were first induced into a deep anesthesia state using 5% isoflurane gas. The hair on the rat’s head was shaved, and the fat and muscle tissue between the scalp and skull were cleaned with a 30% hydrogen peroxide solution to expose the bregma. Based on the brain stereotactic atlas, the hippocampal region of the rat was identified and a 2 × 2 mm window was drilled into the skull. The dura mater was carefully removed to prevent damage to the probes. Figure 8 provides a schematic diagram of the surgical implantation of the probes into the rat hippocampus. Using a micromanipulator, the devices were implanted into the hippocampus at a constant speed of 2 μm/s. The implantation process was paused for 2–3 mins every 200 μm to minimize tissue damage and displacement. Once implanted, the devices were secured to the rat’s brain using dental cement. Additionally, skull screws were inserted into the rat’s skull and grounded to reduce interference during neural signal detection. All animal experiments were carried out with the permission of the Beijing Association on Laboratory Animal Care (Beijing, China) and approved by Institutional Animal Care and Use Committee at the Aerospace Information Research Institute, Chinese Academy of Sciences (AIRCAS, Beijing, China).
